# Computational exploration of *Picrasma quassioides* compounds as CviR-mediated quorum sensing inhibitors against *Chromobacterium violaceum*

**DOI:** 10.3389/fchem.2024.1286675

**Published:** 2024-05-28

**Authors:** Prasanna D. Revanasiddappa, Gowtham H. G., Chandana K. P., Shilpa Natarajamurthy, Nataraj K., Sushma Pradeep, Chandan Shivamallu, Gehan M. Elossaily, Raghu Ram Achar, Ekaterina Silina, Victor Stupin, Natalia Manturova, Ali A. Shati, Mohammad Y. Alfaifi, Serag Eldin I. Elbehairi, Amruthesh Kestur Nagaraj, Murali Mahadevamurthy, Shiva Prasad Kollur

**Affiliations:** ^1^ Department of Biotechnology, Siddaganga Institute of Technology, Tumkur, India; ^2^ Department of Studies and Research in Food Science and Nutrition, Karnataka State Open University, Mysuru, India; ^3^ Department of Studies in Microbiology, University of Mysore, Mysore, India; ^4^ Department of Studies in Botany, University of Mysore, Mysore, India; ^5^ Department of Biotechnology and Bioinformatics, School of Life Sciences, JSS Academy of Higher Education and Research, Mysuru, India; ^6^ Department of Basic Medical Sciences, College of Medicine, AlMaarefa University, Riyadh, Saudi Arabia; ^7^ Division of Biochemistry, School of Life Sciences, JSS Academy of Higher Education and Research, Mysuru, India; ^8^ Department of Pathophysiology, I.M. Sechenov First Moscow State Medical University (Sechenov University), Moscow, Russia; ^9^ Department of Hospital Surgery, Pirogov Russian National Research Medical University, Moscow, Russia; ^10^ Biology Department, Faculty of Science, King Khalid University, Abha, Saudi Arabia; ^11^ School of Physical Sciences, Amrita Vishwa Vidyapeetham, Mysuru, India

**Keywords:** Chromobacterium violaceum, CviR protein, *in silico*, Picrasma quassioides, quorum sensing

## Abstract

*Chromobacterium violaceum* an opportunistic human pathogenic bacterium, exhibits resistance to conventional antibiotics by exploiting its quorum sensing mechanism to regulate virulence factor expression. In light of this, disrupting the quorum sensing mechanism presents a promising avenue for treating infections caused by this pathogen. The study focused on using the cytoplasmic quorum sensing receptor CviR from *C. violaceum* as a model target to identify novel quorum sensing inhibitors from *P. quassioides* through *in silico* computational approaches. Molecular docking analyses unveiled that several phytochemicals derived from *Picrasma quassioides* exhibit the potential to inhibit quorum sensing by binding to CviR protein. Notably, the compounds such as Quassidine I (– 8.8 kcal/mol), Quassidine J (– 8.8 kcal/mol), Kumudine B (– 9.1 kcal/mol) and Picrasamide A (– 8.9 kcal/mol) exhibited high docking scores, indicating strong binding affinity to the CviR protein. The native ligand C6-HSL (N-hexanoyl-L-homoserine lactone) as a positive control/co-crystal inhibitor also demonstrated a significant binding energy of—7.7 kcal/mol. The molecular dynamics simulation for 200 ns showed the thermodynamic stability and binding affinity refinement of the top-ranked CviR inhibitor (Kumudine B) with its stable binding and minor fluctuations compared to positive control (C6-HSL). Pharmacokinetic predictions indicated that Kumudine B possesses favourable drug-like properties, which suggest its potential as a drug candidate. The study highlight Kumudine B as a potential agent for inhibiting the CviR protein in *C. violaceum*. The comprehensive evaluation of Kumudine B provides valuable insights into its pharmacological profiles, facilitating its assessment for diverse therapeutic applications and guiding future research activities, particularly as antibacterial agents for clinical drug development.

## 1 Introduction

Numerous bacteria employ the quorum sensing system to communicate among themselves and synchronize their actions according to cell density (Rutherford and Bassler, 2012; Zhou et al., 2020). This intricate mechanism allows bacterial populations to collectively regulate gene expression, thereby leading to the activation or repression of certain traits or behaviours. Quorum sensing allows bacteria to perform various activities, including virulence factor expression, biofilm formation, pathogenicity, competition, antibiotic resistance and nutrient utilization (Swem et al., 2009). Thus, bacteria must perceive alterations in their surrounding environment and adapt their responses accordingly. Quorum sensing inhibition involves the disruption of bacterial communication pathways, potentially leading to the reduction of both virulence and biofilm formation and other behaviours regulated by quorum sensing. Quorum sensing inhibitors have gained attention as potential alternatives to traditional antibiotics, which may lead to antibiotic resistance reduction by targeting bacterial communication instead of killing the bacteria. Additionally, inhibiting quorum sensing can attenuate the virulence factor expression, rendering bacteria less harmful and therefore, *Chromobacterium violaceum* based assays can aid in identifying compounds with anti-virulence and therapeutic potential.

*Chromobacterium violaceum* is an aggressive, opportunistic bacterial pathogen that is capable of instigating pneumonia, urinary tract infection, endocarditis, fulminant sepsis, liver abscesses, meningitis, and hemophagocytic syndrome in humans when entering the bloodstream via the wounds (Kothari et al., 2017; Venkatramanan et al., 2020; Alisjahbana et al., 2021). Inside the *C. violaceum* cell, the N-acylhomoserine lactone (AHL) molecules are synthesized by LuxI synthase enzyme, which catalyzes the formation of AHL molecules from the precursors acyl-acyl carrier protein (acyl-ACP) and S-adenosyl-L-methionine (SAM) through the addition of homoserine lactone ring. Once synthesized, the AHL molecules are immediately diffused from the bacterial cell into the surrounding environment and are able to diffuse back into the cell through the extracellular space. The AHL molecules that have subsequently diffused back encounter and interact with a specific CviR receptor protein, which is generally located on the surface of *C. violaceum* cells. The interaction leads to the binding of CviR-AHL complex to a specific promoter region, ultimately resulting in the gene activation governed by the quorum sensing system (Venkatramanan et al., 2020; Dimitrova et al., 2023). Overall, *C. violaceum* is is an interesting model organism for studying various biological processes, including quorum sensing, pigment production, and the interactions between bacteria and their environments. Indeed, the quorum sensing system of *C. violaceum* has been extensively employed as a model for studying the potential inhibition of quorum sensing by natural compounds and synthetic molecules.

It is evident that natural products hold great promise for drug discovery, and their potential as quorum sensing inhibitors against *C. violaceum* is a fascinating avenue of research (Rasoanaivo et al., 2011; Singh and Bhatia, 2018; Venkatramanan et al., 2020). Over 80% of drugs have origins in natural products and their derivatives, highlighting the importance of this approach. Numerous plant extracts and natural compounds have demonstrated the ability to inhibit quorum sensing in *C. violaceum* (Bouyahya et al., 2017; Moradi et al., 2020; Dimitrova et al., 2023). These natural compounds may interfere with bacterial communication by mimicking or competing with signalling AHL molecules commonly used in quorum sensing systems. Some natural molecules produced by plants (such as furanone) exhibit structural similarities to AHL molecules, considered AHL mimic compounds (Pérez-Montaño et al., 2013). This structural similarity allows them to bind to the same receptor sites on bacterial proteins involved in quorum sensing, particularly the LuxR type transcription factor CviR in *C. violaceum* (Kothari et al., 2017). These natural compounds can competitively hinder the binding of native AHL molecules through their interaction with these sites, thereby disrupting the quorum sensing process. The LuxR type transcription factor CviR plays an essential role in the quorum sensing system of *C. violaceum*. It serves as a receptor for AHL molecules and its activation triggers downstream gene expression events that contribute to the coordinated behaviours of the bacterial population.

*Picrasma quassioides*, also known as Japanese bitter ash or bitterwood, has been used in traditional herbal medicine in some Asian cultures for its potential medicinal properties (Guo et al., 2019). This plant is used as antioxidant, anti-inflammatory, antimicrobial, antitumor and gastroprotective that support liver health and function (Mohd Jamil et al., 2020; Lee et al., 2021). However, it is crucial to highlight that scientific research on its medicinal effects is limited, and many of its traditional uses have not been extensively studied or validated through rigorous studies. The literature indicates that *C. violaceum* exhibits significantly higher resistance to a wide range of beta-lactam antibiotics, such as penicillin, ampicillin, and cephalosporin, primarily attributed to its heightened beta-lactamase activity (Yang and Li, 2011). It is also worth emphasizing that further research is essential to identify potential quorum sensing inhibitors against *C. violaceum* and advance them to the subsequent stages of drug discovery. Computational modelling is pivotal in drug discovery and development as it helps researchers quickly identify and optimize potential drug candidates from large compound libraries more efficiently and cost-effectively. Hence, the objective of the present study was to employ computational modelling to identify potential plant-based quorum sensing inhibitors from *P. quassioides* targeting CviR in *C. violaceum*.

## 2 Materials and methods

### 2.1 Preparation of ligands

The 44 phytochemicals extracted from *P. quassioides* were used as the ligands in virtual screening against the quorum sensing target CviR protein (Mohd Jamil et al., 2020). The native ligand, C6-HSL (N-hexanoyl-L-homoserine lactone-an AHL standard), which is complexed with the CviR protein served as a positive control (a co-crystal inhibitor) for comparative analysis in this study (Yin et al., 2012). The structured data format (SDF) files containing both two dimensional (2D) and three dimensional (3D) chemical structures for the chosen ligands were acquired from the PubChem database (Kim et al., 2019). The chemical structures that were unavailable in the database were drawn with the MarvinSketch software (version 18.3.0). The OpenBabel software (version 2.3.2) was used to convert the SDF files into the Protein Data Bank (PDB) file format. Before conducting molecular docking, the geometry of the ligand structures was optimized by employing the PRODRG online server, which ensures that the ligand structures are in their most energetically favourable conformations for the docking calculations.

### 2.2 Preparation of target protein structure

The crystal structure of CviR from *C. violaceum* that bound to C6-HSL (PDB: 3QP6 at a resolution of 2.00 Å) was obtained from the Research Collaboratory for Structural Bioinformatics (RCSB) PDB database in PDB format and considered as a quorum sensing target protein receptor (Chen et al., 2011). The Modeller software (version 10.4) was used to process the protein structure first by deleting unnecessary water molecules, inserting hydrogen atoms, adding back the missing residues and assigning appropriate charges. After Modeller processing, the energy minimization was accomplished using the Swiss PDB viewer software (version 4.1.0) to relax the structure and remove steric clashes. Before proceeding with protein-ligand docking, the processed protein structure was further validated to ensure its quality and reliability.

### 2.3 Molecular docking analysis

The active site of CviR protein was ascertained by utilizing CASTp 3.0 web server (Tian et al., 2018). The AutoDock Vina 1.2.0 implicated in PyRx 0.8 virtual screening software was exploited to carry out the docking of numerous ligands simultaneously (Eberhardt et al., 2021). The cubical grid with fixed dimensions of 18.85 × 19.21 × 19.35 Å in the xyz directions and 0.375 Å in the grid spacing was used around the native ligand binding pocket of the CviR protein. The docking exhaustiveness was configured to its default value of 100 and the binding energy, usually expressed in kilocalories per mole (kcal/mol), was used to calculate the docking scores. The conformation of molecules with the lowest binding energy was selected as the most favourable docked pose for further investigation. BIOVIA Discovery Studio Visualizer (version 4.5) analyzed the best-docked complex’s protein-ligand interaction. To further validate the reliability of the docking process, re-docking was conducted using the best compounds and CviR protein.

### 2.4 Molecular dynamics simulation

The MD simulation was performed to know the ligand’s interactions, stability, and behaviour within the enzyme’s active site using the GROMACS 2020.6 simulation software package with GROMOS 54A7 force field (Altharawi et al., 2021). The CviR protein complexed with Kumudine B (the top potential compound) of *P. quassioides* and positive control were selected for MD simulation to confirm the docked protein-ligand complexes’ structural stability and binding mode. The selected ligand molecules’ topology was produced using the Automated Force Field Topology Builder (ATB) and Repository server (Malde et al., 2011). The missing residues (*viz.,* MET1, VAL2, THR3, SER4, LYS5, PRO6, PRO52, SER79, and GLY265) were added to the protein structure using MODELLER 10.4 software. The protein topology file was generated by pdb2gmx module of GROMACS with GROMOS 54A7 force field. The complexes were solvated by the Simple Point Charge (SPC) intermolecular water model in a cubic box with water molecules extending 10 Å from the protein on all sides. The counter amount of 0.15 M salt (Na^+^ and Cl^−^ions) was added to neutralize the charge of the system (Chirasani et al., 2016; Keshavamurthy et al., 2023; Revanasiddappa, 2023). The energy of the neutralized solvated system was minimized by employing the steepest descent method for 50,000 steps to relax the system to a stable starting conformation. After energy minimization, the resulting system was equilibrated in a constant number of atoms, volume, and temperature (NVT ensemble) and a constant number of atoms, pressure, and temperature (NPT ensemble) conditions for 0.1 ns. The system was gradually heated to 310 K using a velocity rescaling (v-rescale) thermostat with a 0.1 ps temperature coupling constant and the pressure was maintained at 1 bar using a Parrinello-Rahman barostat with isotropic pressure coupling. The long-range electrostatic interactions in systems were managed using the Particle Mesh Ewald (PME) approach with a threshold of 0.1 nm. The Linear Constraint Solver (LINCS) method restrained or constrained all bonds containing hydrogen atoms. Finally, the MD simulation was conducted for 200 ns at 310 K temperature and 1 bar constant pressure with the time step size of 2 fs. The study’s parameters used to perform MD simulation are provided in Supplementary Table S1. The trajectories generated from MD simulations were the root mean square deviation (RMSD), root mean square fluctuation (RMSF), radius of gyration (Rg), solvent-accessible surface area (SASA) and number of hydrogen bonds formed between the protein and ligand to reveal potential interactions. The Xmgrace tool was used to plot the graphs to visualize the results of generated MD trajectories.

### 2.5 Binding free energy calculation

Molecular Mechanics/Poisson-Boltzmann Surface Area (MM-PBSA) method was employed for estimating the binding free energy of a ligand to a protein based on MD simulations (Kumari et al., 2014). Both gmmpbsa software and MmPbStat.py script utilized the GROMACS 2020.6 trajectories to calculate binding free energy for each protein-ligand complex. The g_mmpbsa program was exploited to compute a ligand’s overall binding free energy (∆G) when bound to a protein in a solvent environment by considering the molecular mechanics energy, solvation energies, and entropy. The calculation used the stable MD trajectories extracted from the 200 ns trajectory. Eqs 1, 2 from Martiz et al. (2022) were employed to compute the free binding energy.

$$∆G_{\text{Binding}}=G_{\text{Complex}}\;–\;\left(G_{\text{Protein}}+G_{\text{Ligand}}\right)$$ (1)

$$∆G=∆E_{\text{MM}}+∆G_{\text{Solvation}}\;–\;T∆S=∆E_{\left(\text{Bonded}+\text{Non}-\text{bonded}\right)}+∆G_{\left(\text{Polar}+\text{Non}-\text{polar}\right)}\;–\;T∆S$$ (2)

G_Binding_: Binding free energy; G_Complex_: Total free energy of protein-ligand complex; G_Protein_ and G_Ligand_: Total free energies of protein and ligand in solvent, respectively; ∆G: Standard free energy change; ∆E_MM_: Average molecular mechanics potential energy in vacuum; ∆G_Solvation_: Solvation energy contribution to the free energy change; ∆E: Total energy of bonded and non-bonded interactions; ∆S: Change in entropy upon ligand binding and T: Temperature in Kelvin.

### 2.6 Absorption, distribution, metabolism, excretion, and toxicity analysis

The pkCSM signature was utilized to compute the physicochemical descriptors and pharmacokinetic properties of the most promising compounds (Pires et al., 2015). The physicochemical descriptors consisted of molecular weight, partition coefficient (Log P), number of rotatable bonds, number of hydrogen bond acceptors and donors, and topological polar surface area (TPSA).

## 3 Results and discussion

### 3.1 Molecular docking analysis

**FIGURE 1:**
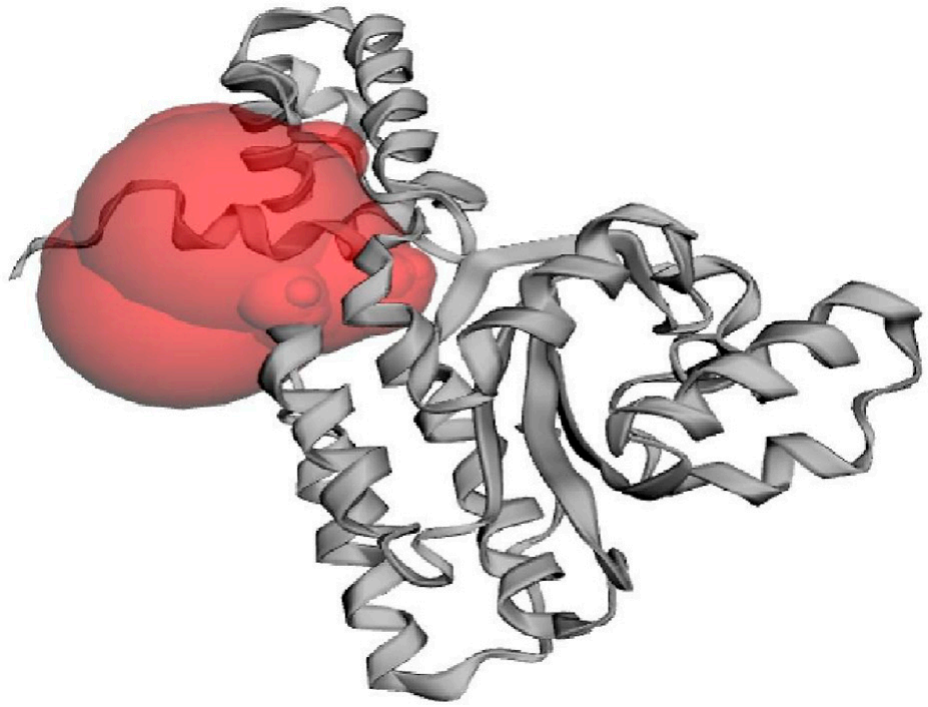
The active site of CviR protein (PDB: 3QP6) of *C. violaceum* as predicted from CASTp 3.0 web server.

Virtual screening encompasses utilizing computational techniques to examine extensive compound databases to forecast their ability to bind to a specific target protein and helps to identify the molecules that can act as drugs (Gowtham et al., 2023). Techniques like molecular docking, where the 3D structures of compounds are docked into the ligand binding site of a given target protein, are commonly used in virtual screening (Murali et al., 2022a). The CviR protein recognizes and interacts with the AHL molecules produced by *C. violaceum* to ensure that *C. violaceum* responds to its signalling molecules and not those produced by other bacterial species. In addition, the ligand-binding domain found in the CviR protein structure is responsible for the recognition and binding of AHL molecules. The CviR protein also plays an important role in quorum sensing regulation by regulating the expression of genes associated with *C. violaceum*’s pathogenicity and hence it is a potential target to prevent quorum sensing and reduce bacterial pathogenicity (Dimitrova et al., 2023). The native ligand binding N-terminal domain of the CviR protein (PDB: 3QP6) contains the active site residues of ILE57, VAL59, MET72, TYR80, TRP84, LEU85, TYR88, ASP97, ILE99, MET100, TRP111, PHE115, PHE126, ALA130, MET135, ILE153, and SER155 which are found responsible for binding of ligands on to the target protein. Figure 1 demonstrates the active site of CviR protein where it interacts with other molecules, typically its substrates, to catalyze chemical reactions and perform its biological function.

**FIGURE 2:**
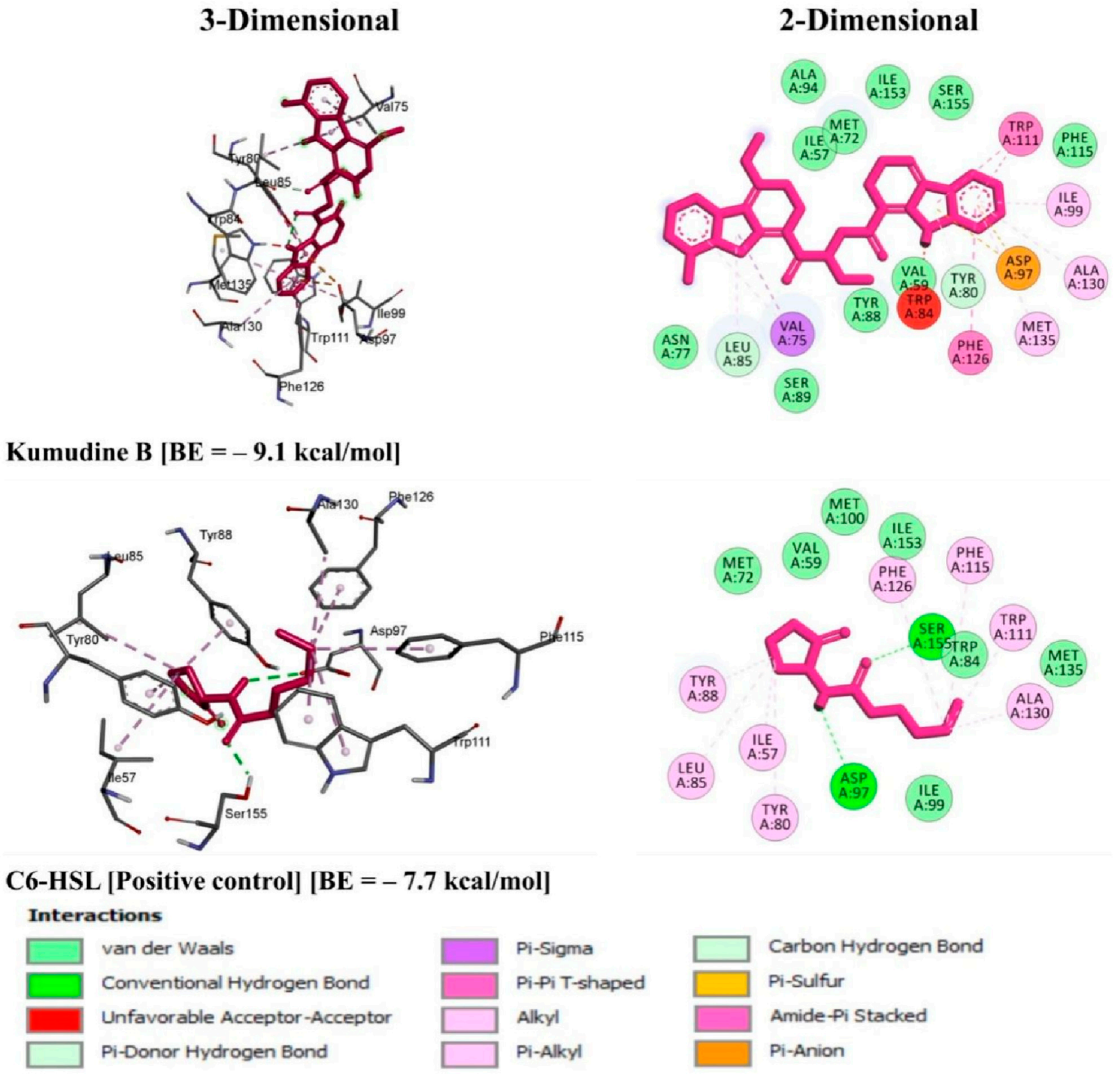
3D and 2D illustrations of *C. violaceum* CviR protein receptor (PDB: 3QP6) interaction with the top potential phytochemical from *P. quassioides* and positive control.

It is well noted that the docking scores provide insights into the binding affinity of these compounds to the target protein, with lower scores typically indicating stronger binding (Anandan et al., 2022). Some of these phytochemicals, such as Quassidine I (– 8.8 kcal/mol), Quassidine J (– 8.8 kcal/mol), Kumudine B (– 9.1 kcal/mol) and Picrasamide A (– 8.9 kcal/mol) displayed high docking scores, suggesting their strong binding affinity to the CviR protein (Table 1; Supplementary Table S2 **)**. The C6-HSL used as a positive control, also demonstrates a notable docking score of—7.7 kcal/mol. The docking scores indicate the potentiality of these phytochemicals from *P. quassioides*, to interact with the CviR protein in *C. violaceum.* It was noticed that the selected ligands were found to bind at the native ligand-binding site of the CviR protein, which had the potential to inhibit the protein function (Figure 2; Supplementary Figure S1). The strong binding affinity displayed by the identified phytochemicals, as reflected in their favorable binding energy values, can be rationalized by considering various factors (such as complementary molecular interactions, hydrophobic interactions, good shape and size complementarity, solvation effects, specific binding sites on the protein, multiple binding modes within the binding site, and structural features of the ligands) that contribute to the stability of the complexes (Agu et al., 2023; Mohanty and Mohanty, 2023). The classification of hydrogen bond interactions based on the specified distance criteria from Imberty et al. (1991) provides a detailed overview of the strength and nature of hydrogen bond interactions between these compounds and enzymes. A detailed summary of the binding interactions established between top ligand and residues of CviR protein is provided in Supplementary Table S3. Compounds with higher negative scores suggest stronger binding interactions and may hold promise for further investigation as potential antimicrobial agents against *C. violaceum*. The re-docking study confirmed that the potential compounds and positive control showed the same binding energy with RMSD values between ≤2.0 Å, which agrees with the findings of Ramírez and Caballero (2018). Recently, Murali et al. (2023) have intended to identify the effective inhibitors of quorum sensing from 123 bioactive compounds of *Cladosporium* spp. against *C. violaceum* through computational studies and noted that six compounds had higher docking score than Azithromycin, suggesting their strong affinity towards the CviR protein. Additional experiments and studies were warranted in the present study to validate these findings and assess the compounds’ efficacy as antimicrobial agents.

**TABLE 1:** Docking scores of best phytochemicals from *P. quassioides* and positive control against CviR protein (PDB: 3QP6) of *C. violaceum.*

Compound name	PubChem CID	Docking score (kcal/mol)
* Quassidine I	–	−8.8
* Quassidine J	–	−8.8
* Kumudine B	–	−9.1
* Picrasamide A	–	−8.9
N-hexanoyl-L-homoserine lactone (C6-HSL) [Native ligand]	CID_10058590	−7.7

^*^The chemical structures that were unavailable in the database were drawn with the MarvinSketch software according to Mohd Jamil et al. (2020) and used in the present study.

### 3.2 MD simulation

**FIGURE 3:**
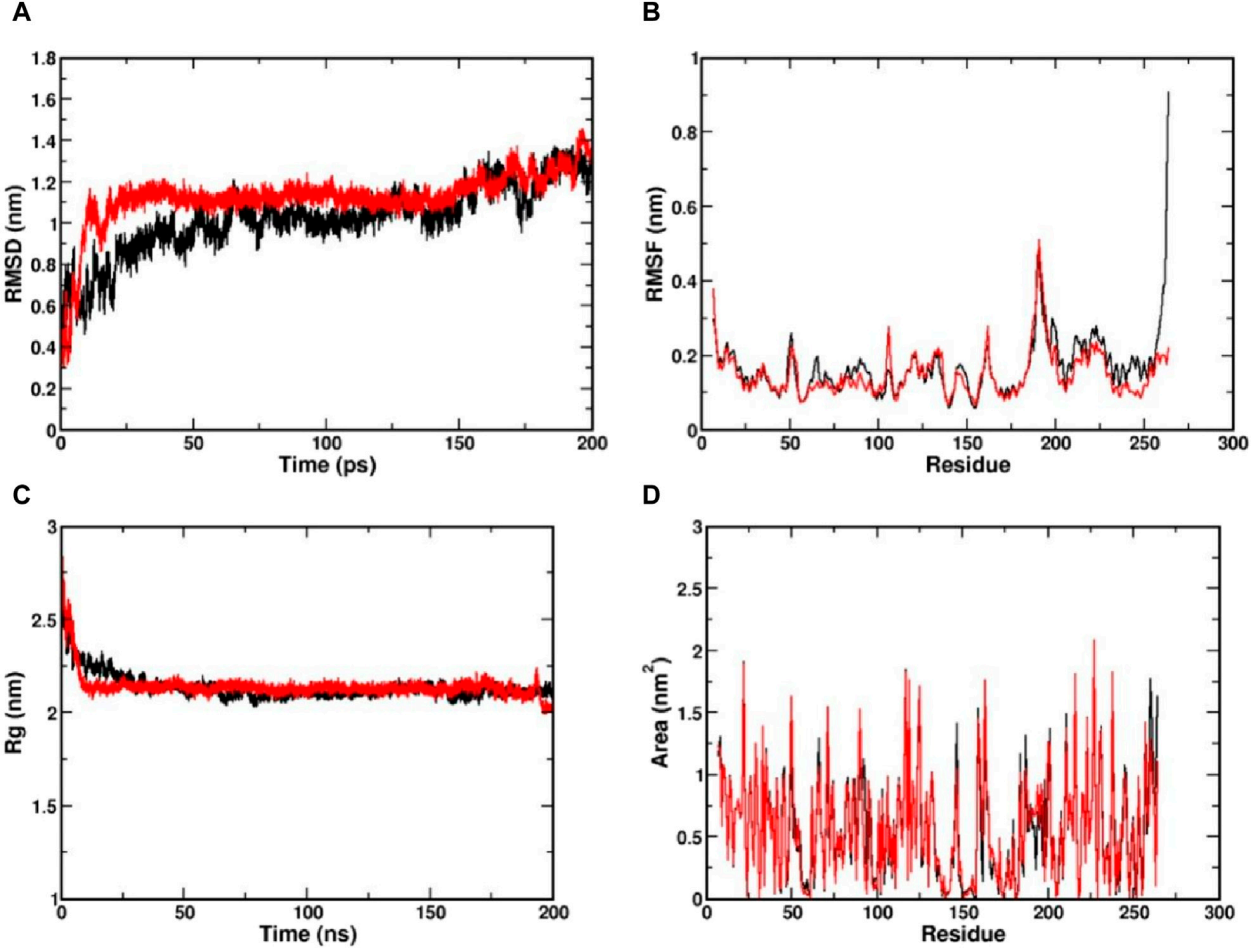
Visualization of MD simulation results of CviR protein complexed with Kumudine B of *P. quassioides* and positive control (C6-HSL) during the simulation of 200 ns. **(A)** RMSD plot, **(B)** RMSF plot, **(C)** RG plot, and **(D)** SASA plot. Black: CviR protein-C6-HSL complex, Red: CviR protein-Kumudine B complex.

The MD simulation is a computational technique exploited to investigate the dynamic behaviour of a protein-ligand complex in a solvated environment (Hospital et al., 2015). The CviR protein complexed with Kumudine B (the top potential compound) of *P. quassioides* and positive control (C6-HSL) were selected for MD simulation. Each simulation was run for 200 ns to understand the protein-ligand complex’s stability, dynamics, and interactions in a solvated environment. In the MD simulation, the RMSD measures the deviation of backbone atoms of the system present in all the frames of the trajectory against the reference structure (Sargsyan et al., 2017). Here, the RMSD of the protein was found to be stable after 25 ns in both complex systems, which indicates that they are well-equilibrated. The average RMSD value for CviR protein complexed with C6-HSL was found to be 1.09 nm, whereas the RMSD of CviR protein complexed with Kumudine B was found to be 1.1 nm (Figure 3A). However, the measured RMSD had slightly higher values than the usual RMSD’s of many other protein complexes. To identify the reason for this, the enzyme structure was indexed into two parts, and the RMSD of the individual segment was measured to conclude. It is noted that the segment consisting of residues between 1 and 185 contributing for ligand binding is considered as an N-terminal domain, while the segment of residues between 201 and 261 was considered as a C-terminal domain. The average RMSD of N-terminal domain was found to be 0.29 nm compared to the average RMSD of 0.3 nm in the case of C-terminal domain (Supplementary Figure S2). The RMSD measured for the N-terminal domain was found to have less deviation from the crystal conformation, indicating its stable conformation. Further, the increased RMSD in the overall complex structure may be due to a long loop region between the domains, making the complex structures deviate more as noted by Kumar et al. (2022). Also, the loop region may not have its principal motion in a specified direction, possibly making the loop fluctuate randomly, which agrees with the findings of Bucio-Cano et al. (2015).

The RMSF is a metric used to analyze individual atoms’ or residues’ flexibility or mobility within a protein structure during MD simulation as it provides insights into protein regions that exhibit significant fluctuations in their positions throughout a simulation that correspond to loop regions, surface-exposed areas, or segments with inherent structural flexibility (Boroujeni et al., 2021). Here, the complex structure with high RMSF indicates a flexible region, while lower RMSF indicates a comparatively stable region. From the RMSF plot (Figure 3B), clear evidence of lower RMSF was observed in the case of Kumudine B between the region 60 and 100. The residue region between 60 and 100 majorly encompasses the active site region, indicating the ability of Kumudine B to bind strongly in the pocket. In addition, the regions between 186 and 200 in the RMSF plot showed a significant rise with a peak value of 0.5 nm in both systems. This observation was directly correlated with the RMSD plot, where the loop region was presumed to vary the stability of the protein, thereby contributing to higher RMSD.

The Rg indicates the compactness of the protein over simulation time. Here, the larger values of Rg indicate a more spread-out or extended molecule, while the smaller values indicate a more compact structure (Lobanov et al., 2008). The Rg values of both simulated protein complexes with Kumudine B and C6-HSL were found to be averaged at 2.21 and 2.22 nm, respectively (Figure 3C). As no significant difference in the Rg was observed in the protein complexes, the N-terminal ligand binding domain (as defined during RMSD calculations) alone in both complexes was considered. Here, the Rg of N-terminal domain of C6-HSL bound system was found to be 1.628 nm compared to 1.608 nm in Kumudine B bound system, whereas for C-terminal segment C6-HSL the Rg was found to be 1.157 nm and Kumudine B bound system was found to be 1.121 nm (Supplementary Figure S3). Here, a slight decrease in Rg was reported, indicating the differential compactness. Similarly, the SASA of the protein-ligand complex was also performed to assess the accessibility of the solvent molecules, such as water, in a solution (Ali et al., 2014). The SASA analysis provides insights into the complex’s interactions with its surrounding environment and can be used to study conformational changes, binding events, and other dynamic behaviors. Here, the SASA plot (Figure 3D) showed a similar trend to Rg, where SASA values appear nearly identical in both complexes. Therefore, the N-terminal ligand binding domain SASA was calculated for both systems (Supplementary Figure S4). From the plot, it is evident that the SASA for Kumudine B and C6-HSL bound systems were found to be nearly the same with slight variations. Cumulatively, by observing the results of Rg and SASA, it is proposed that the Kumudine B binding initiates conformational change in the CviR protein, making the structure have differential compactness, presenting a slightly varied surface area for the solvent to interact, resulting in differential SASA values. The results possibly make Kumudine B to bind strongly in the active pocket site of the CviR protein. Thus, the binding interactions of the residues of the CviR protein with the ligands to unwrap the deep understanding of the energetically favourable environment are discussed in the next section.

### 3.3 Binding free energy calculation

**FIGURE 4:**
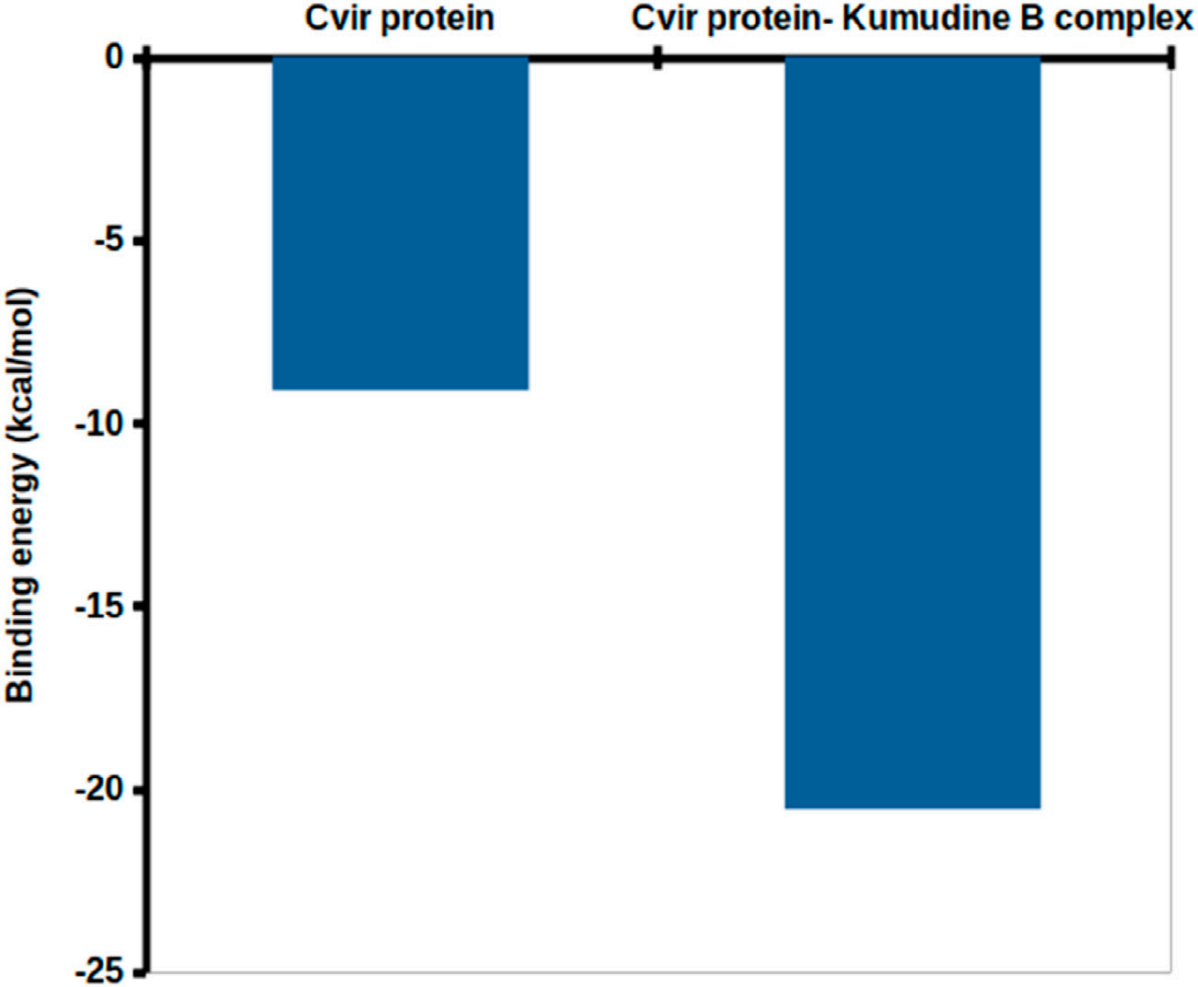
Binding free energy obtained with the MMPBSA analysis.

The binding free energy is a comprehensive measure of the strength of interaction between the protein and ligand, which provides insights into the thermodynamics of protein-ligand interaction that supports drug discovery and design efforts (Kumari et al., 2014). The obtained binding free energy values can be applied to assess the strength of protein-ligand interactions and more negative ∆G values indicate stronger ligand-protein interactions. The most relevant interactions are considered on a stable trajectory of 200 ns simulated data to arrive at the conclusion. The analysis focused on the influence of van der Waals’ energy and binding energies on complex formation, and the results suggest that van der Waals interactions play a significant role in driving the formation of protein-ligand complexes. The results summarized in Table 2 outline the various energy components calculated, including van der Waals energy, electrostatic interactions, solvation energies, etc. The comparison of these energy components reveals the contribution of each term to the overall binding free energy along with the stability and affinity of protein-ligand complexes. The CviR protein-Kumudine B complex had the lowest van der Waals energy (– 41.701 kcal/mol) with less negative electrostatic energy (– 7.353 kcal/mol), than positive control, concluding that it has the strongest van der Waals interactions with strong electrostatic interactions compared to positive control. The lesser positive polar solvation energy (33.055 kcal/mol) of CviR protein-Kumudine B complex indicated that this complex has a significant favorable contribution to the overall binding energy from the solvation of polar groups. The CviR protein-Kumudine B complex with a more negative SASA energy (– 4.567 kcal/mol) shows that the solvent-accessible surface area achieved beneficial interactions. Overall, Kumudine B complexed with the CviR protein has more negative binding energy (– 20.565 kcal/mol) than positive control, thus suggesting that the interaction between Kumudine B and CviR protein is energetically favorable and stable (Figure 4).

**TABLE 2:** Binding free energy calculation of CviR protein complexed with potential phytochemicals from *P. quassioides* and positive control.

Category	CviR protein-C6-HSL complex	CviR protein- Kumudine B complex
	Values (kcal/mol)	
van der Waal’s energy	−29.915	−41.701
Electrostatic energy	−17.263	−7.353
Polar solvation energy	41.009	33.055
SASA energy	−3.126	−4.567
Binding energy	−9.104	−20.565

### 3.4 ADMET analysis

It is important to note that even structurally similar compounds can exhibit different biological activities due to slight variations in their chemical structures. Additionally, these tools can help filter out compounds that lack drug-like properties or possess toxic effects, thereby improving the efficiency of the drug discovery process (Neves et al., 2018). The physicochemical descriptors for the potential compound (Kumudine B) and positive control were analyzed to evaluate their drug-likeness based on established rules and principles in drug discovery (Table 3). Lower molecular weight (<500 g/mol) compounds are generally considered more favourable for transportation, dispersion, and absorption by cell membranes (Yu et al., 2021). The positive Log *p* values suggest that compounds can easily pass through cell membranes and interact with biomolecules, potentially inhibiting metabolic enzymes, as noted by Liu et al. (2011). The acceptable limit for Log *p* values is < 5, as values within this range are predicted to have ideal lipophilicity for crossing pathogen cell membranes. Kumudine B and C6-HSL showed positive Log *p* values, which indicate their potential for passing through cell membranes and interacting with biomolecules. However, Kumudine B had almost similar Log *p* values, while C6-HSL had a relatively lower Log *p*-value, indicating that C6-HSL is less lipophilic than Kumudine B.

**TABLE 3:** Physicochemical descriptors of potential phytochemical Kumudine B of *P. quassioides* and positive control.

Descriptor	Kumudine B	C6-HSL
Molecular weight (g/mol)	494.50	199.25
Log P	4.93	0.99
Rotatable bonds	7	5
Hydrogen bond acceptors	7	3
Hydrogen bond donors	3	1
TPSA (Å^2^)	130.19	55.40

Lipinski’s rule also suggests that successful oral drugs typically have properties such as ≤10 hydrogen bond acceptors, ≤5 hydrogen bond donors and ≤10 rotatable bonds, which are generally favourable for oral drug development and both Kumudine B and C6-HSL met the Lipinski’s criteria for hydrogen bond donors, acceptors, and rotatable bonds. The TPSA is a parameter that correlates with passive drug transport across membranes and predicts properties like Caco-2 permeability, intestinal absorption, and blood-brain barrier (BBB) penetration (Ertl et al., 2000). The TPSA values < 100 Å^2^ are associated with good absorption and membrane permeability, while the values > 140 Å^2^ indicate strong polarity and potential difficulty in absorption. Kumudine B and C6-HSL had different TPSA, thus indicating differences in their polarity and potential for interactions with polar molecules. From the results of Lipinski’s rule, it may be noted that Kumudine B and C6-HSL met Lipinski’s rule of druglikeness.

The prediction of physicochemical descriptors profoundly impacts the pharmacokinetics properties of potential compounds (Murali et al., 2022b; Gowtham et al., 2022). These properties, including ADMET, are crucial for understanding how a drug or compound behaves within the body. Predicting these properties is essential for developing novel drugs with desired biological activities and a higher likelihood of further success in clinical trials. The results of online pkCSM (pharmacokinetics properties) prediction are presented in Table 4. The studied absorption properties include water solubility, colon carcinoma cell line (Caco-2) permeability, human intestinal absorption, P-glycoprotein interactions, and skin permeability. In drug development, the compound’s water solubility is an important factor that affects its bioavailability, while poor water solubility can lead to challenges in formulating the compound into dosage forms and impact its absorption in the body. Therefore, understanding the water solubility of compounds through their LogS value provides insights into their potential to be absorbed and distributed effectively (Bergstrom and Larsson, 2018). More negative log values suggest better water solubility. Kumudine B had better water solubility as it showed more negative log values than the C6-HSL.

**TABLE 4:** Pharmacokinetics properties of the potential compound of *P. quassioides* and positive control.

Properties	Model name	Kumudine B	C6-HSL
**Absorption**	Water solubility (log mol/L)	−2.984	−1.427
	Caco-2 permeability (log P_app_ in 10^−6^ cm/s)	1.765	1.218
	Human intestinal absorption (% Absorbed)	84.472	90.018
	Skin permeability (log Kp in cm/h)	−2.735	−3.447
	P-glycoprotein substrate	No	No
	P-glycoprotein I inhibitor	Yes	No
	P-glycoprotein II inhibitor	Yes	No
**Distribution**	Human VDss (log L/kg)	−0.435	−0.064
	Human fraction unbound (Fu)	0.17	0.6
	BBB permeability (log BB)	−1.629	−0.15
	CNS permeability (log PS)	−3.902	−2.975
**Metabolism**	CYP2D6 substrate	No	No
	CYP3A4 substrate	No	No
	CYP1A2 inhibitor	No	No
	CYP2C19 inhibitor	No	No
	CYP2C9 inhibitor	Yes	No
	CYP2D6 inhibitor	No	No
	CYP3A4 inhibitor	Yes	No
**Excretion**	Total clearance (log mL/min/kg)	0.56	1.49
	Renal OCT2 substrate	No	No
**Toxicity**	AMES toxicity	No	No
	Human Max. tolerated dose (log mg/kg/day)	0.544	0.706
	hERG I inhibitor	No	No
	hERG II inhibitor	Yes	No
	Oral Rat Acute Toxicity (LD_50_) (mol/kg)	2.461	1.978
	Oral Rat Chronic Toxicity (LOAEL) (log mg/kg bw/day)	1.981	2.489
	Hepatotoxicity	Yes	No
	Skin sensitization	No	No
	*T. pyriformis* toxicity (log μg/L)	0.285	−0.02
	Minnow toxicity (log mM)	−2.42	1.94

Similarly, the Caco-2 monolayers are generally used to assess the intestinal permeability and absorption of drugs in humans (Hubatsch et al., 2007). Permeability coefficient (Papp) values above 8 × 10^−6^ cm/s indicate high Caco-2 permeability and complete absorption from the human intestine. Kumudine B and C6-HSL exhibited moderate permeability during the study with Papp values of <2 × 10^−6^ cm/s. Previous research has demonstrated that human intestinal absorption greatly influences the bioavailability of drug candidates during the drug development process (Dahlgren and Lennernäs, 2019). The compounds are often classified based on their fractional absorption percentages, where ≤30% is considered low and >30% is considered high. Kumudine B and C6-HSL indicated excellent intestinal absorption due to their highest absorption percentage. In addition, skin permeability refers to the rate at which chemical substances penetrate the skin’s outermost layer (Chen et al., 2018) and the human skin permeation coefficient (log Kp) is used to quantify the transportation of small molecules through the stratum corneum. More negative log Kp values suggest better skin permeability and *vice versa*. Kumudine B showed the lowest skin permeability as it had a lower negative log Kp value than C6-HSL. The P-glycoprotein is a key transporter responsible for the absorption and excretion of drugs, wherein P-glycoprotein substrates are transported out of cells by the protein, while its inhibitors slow down its efflux activity (Lin and Yamazaki, 2003). Besides, Kumudine B is the P-glycoprotein inhibitor, which prevents P-glycoprotein mediated drug efflux.

The distribution phase of pharmacokinetics involves delivering a drug to its intended target site in the body after it has entered the bloodstream (Kouskoura et al., 2019). This phase is essential for achieving the desired therapeutic effect and determining how the drug is distributed throughout the body. The volume of distribution (VDss) is a theoretical volume representing the drug distribution throughout the body (Smith et al., 2015). A low predicted log VDss value indicates that the drug is primarily confined to the bloodstream and not extensively distributed into tissues, whereas higher VDss suggests a wider distribution of drugs. The fraction unbound (Fu) represents the unbound (free) drug concentration in plasma and available for distribution, which is crucial for evaluating the therapeutic efficacy of drugs and higher values of the same suggest a larger fraction of the drug is free to move within the body (Watanabe et al., 2018). The study noted that the C6-HSL had the highest Fu, which indicates its larger fraction available for distribution. The BBB permeability is a significant consideration in drug development to prevent drugs from entering the brain and causing unwanted psychotropic effects (Pardridge, 2012). Likewise, the log BB values assess a compound’s ability to cross the BBB and positive values suggest better BBB permeability, while negative values indicate limited BBB penetration. From the obtained results, it was observed that Kumudine B and C6-HSL have negative log BB values that suggest limited BBB penetration ability. The Log PS is a pharmacokinetic parameter that directly measures a drug’s capability to penetrate the central nervous system (CNS) (Clark, 2003). Negative log PS values indicate a compound’s limited CNS permeability. The CNS permeability of Kumudine B and C6-HSL was limited because of their negative log PS values.

Drug metabolism and excretion properties specifically focus on how compounds undergo biotransformation, metabolism by cytochrome P450 (CYP450) enzymes, and subsequent excretion from the body (Zanger and Schwab, 2013). Many drugs and lipophilic xenobiotics are metabolized through oxidative biotransformation, primarily by the body’s cytochrome P450 (CYPs) enzymes. These metabolic processes help to facilitate the excretion of drugs and foreign compounds from the body. Kumudine B is an inhibitor of CYP2C9 and CYP3A4 and has the highest total clearance compared to C6-HSL, which suggests their effective clearance from the body. Also, none of Kumudine B, and C6-HSL are specified as substrates for renal organic cation transporter 2 (OCT2), indicating that this renal transporter does not actively transport them from the kidney. Drug excretion is the process of removing drugs from the body, either in their original form or as metabolites resulting from biotransformation and the process occurs through various routes, including hepatic clearance (from the liver and bile ducts) and renal clearance via transporter proteins like OCT2 (Watanabe et al., 2019).

Predicting various toxicity parameters provides important insights into the compound’s potential safety and toxicological profile. The AMES test is an extensively used bacterial bioassay to detect mutagenic effects and identify possible carcinogens (Vijay et al., 2018). Kumudine B didn’t exhibit AMES toxicity and skin sensitization, which suggests that it does not cause mutations in certain strains of bacteria and may be less likely to cause genotoxic effects apart from being less likely to induce allergic reactions on the skin. It has been observed that the hERG (human Ether-à-go-go Related Gene) channels are related to cardiac function and inhibiting these channels can lead to adverse cardiac effects. Moreover, Kumudine B was found to inhibit the hERG channel II, but not hERG channel I when compared to C6-HSL and didn’t violate Lipinski’s RO5 even though it was found to inhibit CYP450 enzymes and hERG channel II with hepatotoxicity effect. It is well noted that increased lipophilicity (LogP) is associated with target promiscuity and toxicity, whereas an increased molecular weight tends to decrease promiscuity (Shirasaka et al., 2013; Li et al., 2016). Besides, CYP inhibition has been correlated with increased molecular weight and lipophilicity (Kiani and Jabeen, 2019; Hakkola et al., 2020). Based on the results, it is well observed that the lipophilicity and molecular weight of Kumudine B fall within the acceptable range, which meets Lipinski’s rule of drug-likeness. Likewise, Zhao et al. (2019) have reported that the experimental results of cytotoxicity assay against two human hepatocellular carcinoma cell lines (such as Hep3B and HepG2) evaluated by the Sulforhodamine B (SRB) assay showed that Kumudine B obtained from the stem of *P. quassioides* had stronger effect in Hep3B cells. Notably, Kumudine B exhibited the most promising and selective inhibitory activity towards Hep3B cells and caused the death of hepatoma cells through apoptosis induction.

## 4 Conclusion

In conclusion, this study underscores the critical importance of targeting the quorum sensing mechanism in addressing infections caused by the opportunistic human pathogen *C. violaceum*, which exhibits remarkable antibiotic resistance. Focusing on the quorum sensing receptor, CviR, as a model target, we employed innovative *in silico* computational approaches to identify potential quorum sensing inhibitors from *P. quassioides*. Our molecular docking results demonstrated that several phytochemicals from *P.* quassioides such as Quassidine I (– 8.8 kcal/mol), Quassidine J (– 8.8 kcal/mol), Kumudine B (– 9.1 kcal/mol) and Picrasamide A (– 8.9 kcal/mol) possess significant anti-quorum sensing potential, as evidenced by their strong binding affinity to CviR protein with high docking scores compared to the positive control (C6-HSL). Of particular interest was Kumudine B, which emerged as a top drug candidate, consistently outperforming the positive control by binding at the native ligand-binding site of the CviR protein, which had the strong potential to inhibit the protein function. Subsequent thermodynamic stability assessment through a 200 ns MD simulation further supports the potential of Kumudine B as an effective inhibitor with stable binding and minor fluctuations. Moreover, the predicted pharmacokinetic properties of Kumudine B suggest its suitability as a drug candidate, promising future development. In light of the findings of the *in silico* studies, the top-ranked compound from *P. quassioides*, notably Kumudine B, holds considerable potential as *C. violaceum* CviR inhibitor. Its development into a broad-spectrum antibacterial agent is an exciting prospect that depends upon further preclinical and clinical research.

## Data Availability

The raw data supporting the conclusion of this article will be made available by the authors, without undue reservation.
