# Supplementary material for: Computational exploration of Picrasma quassioides compounds as CviR-mediated quorum sensing inhibitors against Chromobacterium violaceum
Source: Front Chem. 2024 May 28;12:1286675. doi: 10.3389/fchem.2024.1286675 (PMC11167448; doi:10.3389/fchem.2024.1286675)
Supplement: Supplementary file 1 [file DataSheet1.docx]

**Supplementary Table 1.** MD simulation parameters of the study.

| **System** | **Total number of atoms** | **Na^+^ ions** | **Cl^–^ ions** | **Force field** | **MD run** |
| --- | --- | --- | --- | --- | --- |
| CviR protein-C6-HSL complex | 192468 | 178 | 182 | gromos54a7_atb.ff | 200 ns |
| CviR protein-Kumudine B complex | 192503 | 178 | 182 | gromos54a7_atb.ff | 200 ns |

**Supplementary Table 2.** Docking scores of phytochemicals from *P. quassioides*and positive control against CviR protein (PDB: 3QP6) of *C. violaceum.*

| **Sl. No.** | **Compound Name** | **PubChem CID** | **Docking score (kcal/mol)** |
| --- | --- | --- | --- |
| 1. | Bruceantin | CID_5281304 | – |
| 2. | * Kumulactone A | – | – |
| 3. | * Kumulactone B | – | – |
| 4. | * Picrasinoside J | – | – 3.2 |
| 5. | * Picrasinoside K | – | – 3.1 |
| 6. | Quassine | CID_65571 | – |
| 7. | Neoquassine | CID_72964 | – 0.9 |
| 8. | Simalikalactone D | CID_6711208 | – |
| 9. | Simalikalactone E | CID_53324651 | – |
| 10. | Picrasin A | CID_185611 | – |
| 11. | Picrasin B | CID_12313355 | – 1.1 |
| 12. | Picrasin C | CID_182145 | – |
| 13. | * 12-hydroxyquassin | – | – 0.6 |
| 14. | Nigakihemiacetal A | CID_441803 | – |
| 15. | Nigakilactone A | CID_10452259 | – |
| 16. | Nigakilactone B | CID_12313347 | – |
| 17. | * 1-hydroxymethyl-8-hydroxy-beta-carboline | – | – 7.4 |
| 18. | Dehydrocrenatidine | CID_5318875 | – 6.5 |
| 19. | * Quassidine I | – | – 8.8 |
| 20. | * Quassidine J | – | – 8.8 |
| 21. | Picrasidine I | CID_5324360 | – 7.5 |
| 22. | * 6,12-dimethoxy-3-ethyl-beta-carboline | – | – 8.1 |
| 23. | * Kumudine A | – | – 7.7 |
| 24. | * Kumudine B | – | – 9.1 |
| 25. | Nigakinone | CID_5320161 | – 6.2 |
| 26. | Methyl nigakinone | CID_638215 | – 6.1 |
| 27. | Picrasidine O | CID_5320558 | – 4.7 |
| 28. | * 4,5-dimethoxy-10-hydroxycanthin-6-one | – | – 6.6 |
| 29. | * 8-hydroxycanthin-6-one | – | – 6.5 |
| 30. | * Picrasmalignan A | – | – 2.9 |
| 31. | Dehydrodiconiferyl alcohol | CID_5372367 | – 6.2 |
| 32. | Picraquassioside C | CID_10077272 | – 3.8 |
| 33. | * Picraquassin A | – | – |
| 34. | * Picraquassin B | – | – |
| 35. | * Picraquassin C | – | – |
| 36. | * Picraquassin D | – | – |
| 37. | * Picraquassin E | – | – 2.9 |
| 38. | * Picraquassin I | – | – |
| 39. | * Picraquasssin J | – | – |
| 40. | * Picraquassin K | – | – 4.6 |
| 41. | * Kumuquassin A | – | – 1.1 |
| 42. | * Kumuquassin B | – | – 2.1 |
| 43. | * Kumuquassin C | – | – 0.3 |
| 44. | * Picrasamide A | – | – 8.9 |
| 45. | N-hexanoyl-L-homoserine lactone (C6-HSL) [Native ligand] | CID_10058590 | – 7.7 |

*The chemical structures that were unavailable in the database were drawn with the MarvinSketch software according to Mohd Jamil et al. (2020) and used in the present study.

**Supplementary Table 3.** Binding interactions of phytochemicals from *P. quassioides* and positive control with CviR protein (PDB: 3QP6) of *C. violaceum.*

| **Compound** | **Interaction type** | **Hydrogen bond**  **interaction with distances** | **Other binding interactions withdistances** |
| --- | --- | --- | --- |
| Kumudine B | **–** | **–** | Pi-Pi: TRP111 (5.68 Å), TRP111 (7.81 Å), PHE126 (7.33 Å)  Pi-Alkyl: ILE99 (5.21 Å), ALA130 (7.32 Å), MET135 (6.13 Å)  Pi-Sigma: VAL75 (4.58 Å), VAL75 (4.63 Å)  Unfavorabledonar-donar: TRP84 (4.60 Å)  Pi-Anion: ASP97 (5.86 Å), ASP97 (6.49 Å)  Carbon hydrogen bond: TYR80 (5.84 Å)  Pi-donar hydrogen bond: LEU85 (3.58 Å), LEU85 (4.89 Å)  van der Waals: ILE57, VAL59, MET72, ASN77, TYR88, SER89, ALA94, PHE115, ILE153, SER155 |
| C6-HSL | Weak | ASP97 (5.56 Å) | Alkyl: ILE57 (5.68 Å), TYR80 (5.52 Å), LEU85 (5.48 Å), TYR88 (4.26 Å), TRP111 (4.86 Å), PHE115 (6.66 Å), PHE126 (5.99 Å), ALA130 (5.12 Å)  van der Waals: VAL59, MET72, TRP84, ILE99, MET100, MET135, ILE153 |
|  | Average | SER155 (3.90 Å) |  |

**
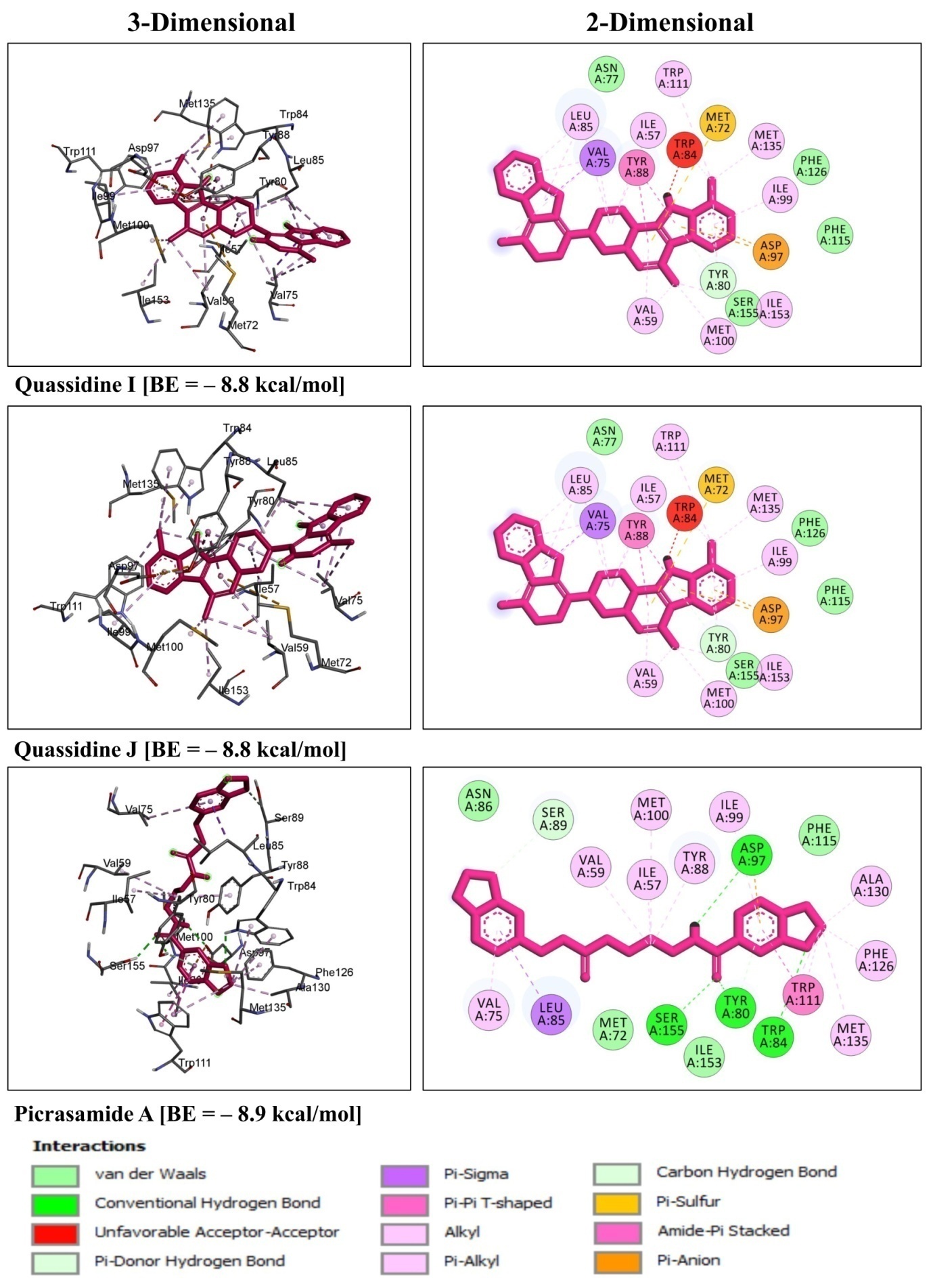
**

**Supplementary Figure 1.** 3D and 2D illustrations of *C. violaceum*CviR protein receptor (PDB: 3QP6) interaction with the phytochemical from *P. quassioides.*

**
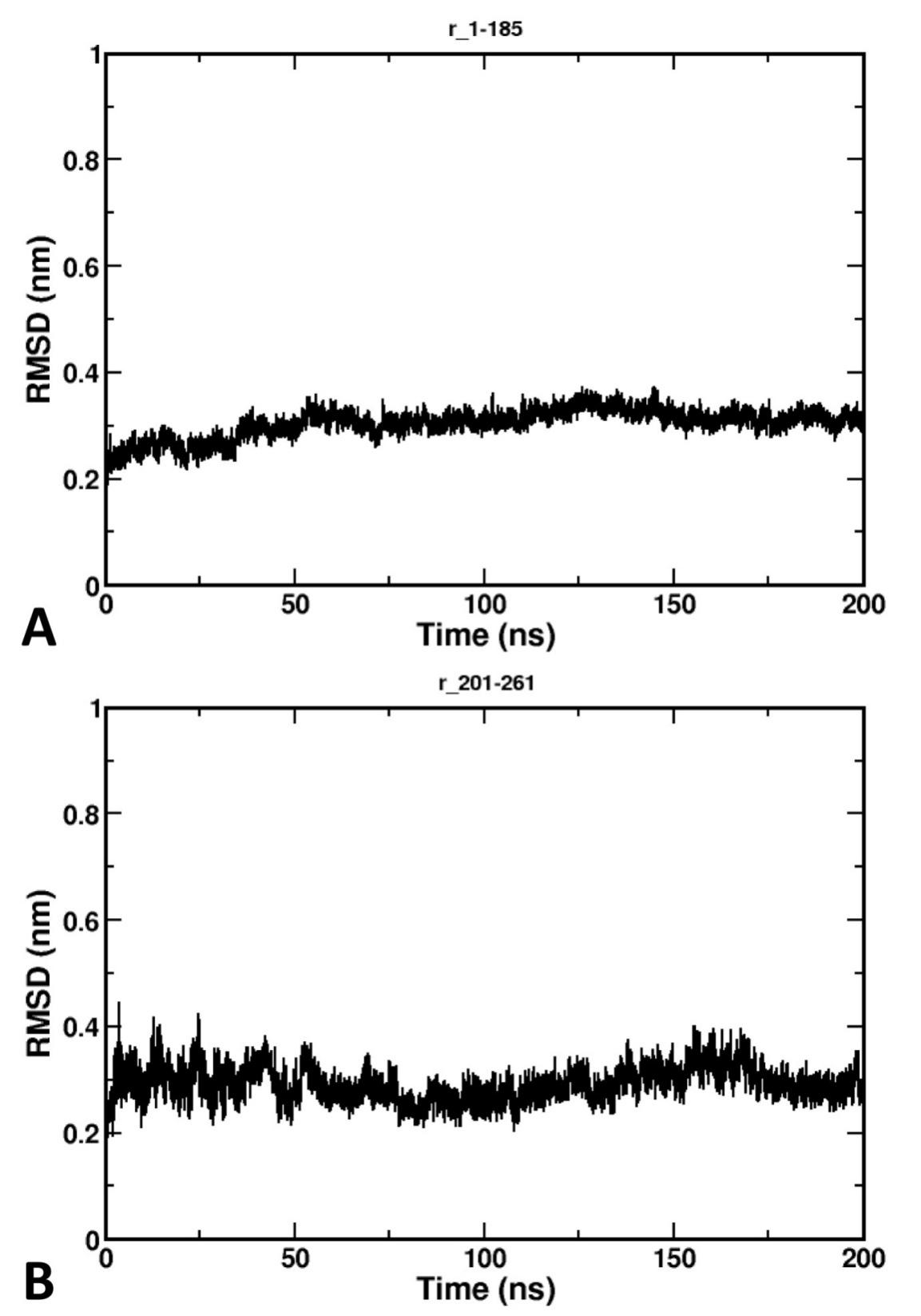
**

**Supplementary Figure 2.** Individual RMSD of the ligand binding N-terminal domain segment (A) and C-terminal domain segment (B) of the CviR protein.


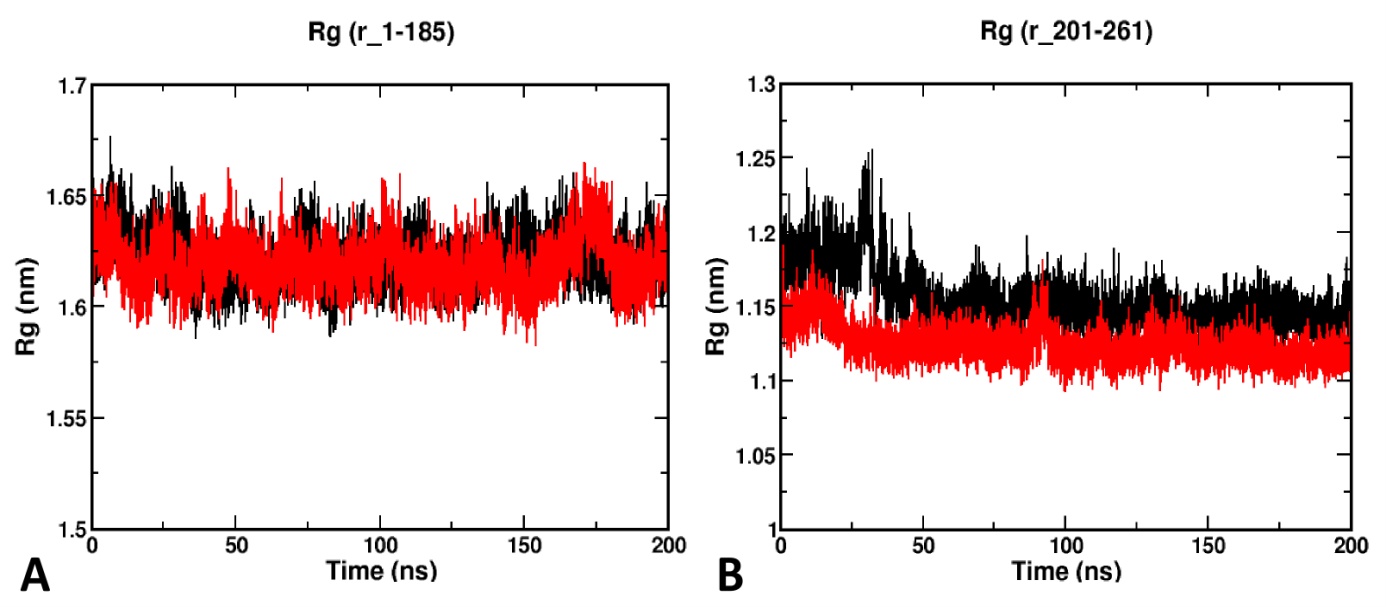


**Supplementary Figure 3.** Individual Rg of the ligand binding N-terminal domain segment (A) and C-terminal domain segment (B) of the CviR protein.


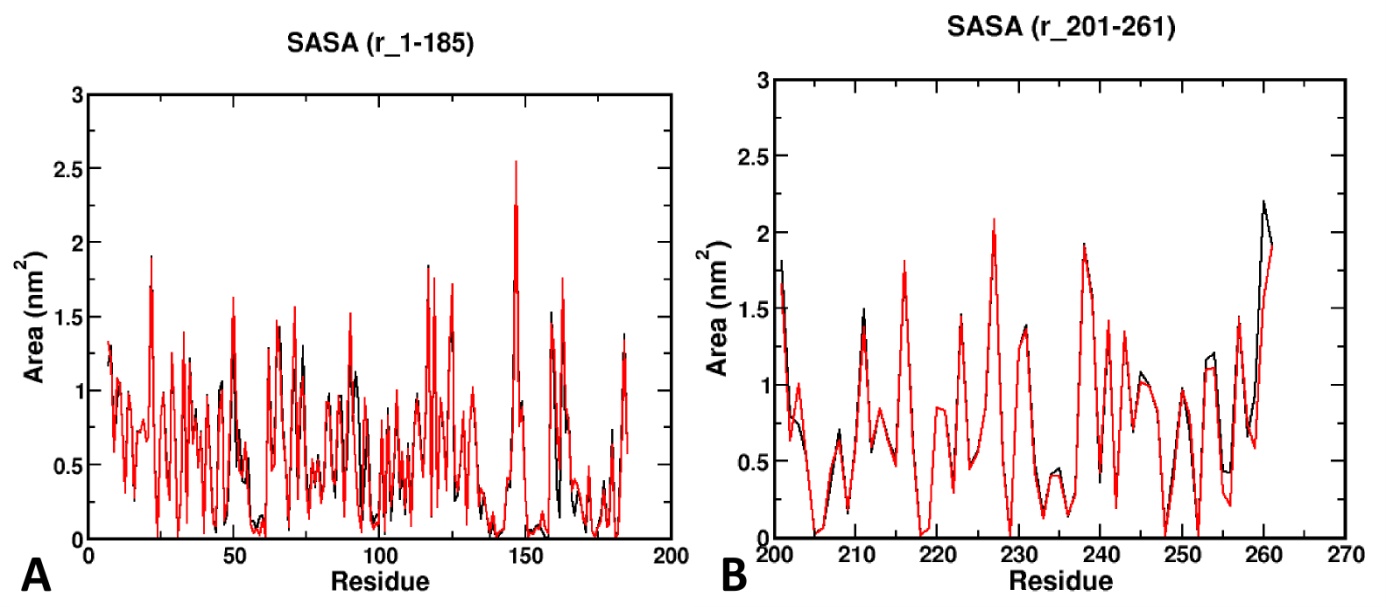


**Supplementary Figure 4.** Individual SASA of the ligand binding N-terminal domain segment (A) and C-terminal domain segment (B) of the CviR protein.
